# A Multidimensional Muscle Quality Index in Severe Obesity: Relationships with Ageing, Adiposity, and Functional Performance

**DOI:** 10.3390/jcm15176696

**Published:** 2026-08-29

**Authors:** Anelise Sonza, Aline Faquin, Gabriella Tringali, Roberta De Micheli, Stefano Lazzer, Alessandro Sartorio

**Affiliations:** 1 Centro de Ciências da Saúde e do Esporte (UDESC/CEFID), Laboratório de Desenvolvimento e Controle Postural (LADESCOP), Universidade do Estado de Santa Catarina (UDESC), Florianópolis 88080-350, SC, Brazil; aline.faquin@udesc.br; 2 Experimental Laboratory for Auxo-Endocrinological Research, Istituto Auxologico Italiano, Istituto di Ricovero e Cura a Carattere Scientifico (IRCCS), Piancavallo-Verbania, 28824 Oggebbio, VB, Italy; g.tringali@auxologico.it (G.T.); r.demicheli@auxologico.it (R.D.M.); sartorio@auxologico.it (A.S.); 3 Department of Medicine, School of Sport Sciences, University of Udine, 33100 Udine, UD, Italy; stefano.lazzer@uniud.it

**Keywords:** muscle quality, severe obesity, handgrip strength, functional power, ageing

## Abstract

**Background/Objectives**: Traditional anthropometric indicators such as body mass index (BMI) may inadequately capture functional muscular impairment in obesity. To investigate a multidimensional composite muscle quality index (MQI) integrating relative strength and lower-limb functional power in adults with severe obesity. **Methods**: The study included 202 adults with severe obesity. The Composite MQI was calculated as the mean of standardized z-scores derived from handgrip strength (HG) normalized by fat-free mass (FFM) (HG/FFM) and sit-to-stand power (STS power) normalized by skeletal muscle mass (MM) (STS power/MM). Associations between the Composite MQI and demographic, anthropometric, and body composition variables were assessed using correlation analyses and multivariable linear regression. Participants were additionally stratified into quartiles of the Composite MQI to explore age-related differences. **Results**: The mean age of the study group was 44.9 ± 14.0 years, and the BMI was 42.5 ± 5.1 kg/m^2^. Age showed a moderate inverse association with the Composite MQI (r = −0.41, *p* < 0.001), whereas BMI showed no significant correlation (r = −0.09). In multivariable regression models adjusted for sex and BMI, increasing age remained independently associated with lower Composite MQI (β = −0.022, *p* < 0.001), whereas BMI was not independently associated with Composite MQI (*p* = 0.373). Men exhibited higher relative and absolute HG than women, although no significant sex differences were observed for STS power/MM. Quartile analyses showed lower Composite MQI values among older participants. **Conclusions**: Among adults with severe obesity, age was associated with lower values of a multidimensional Composite MQI, whereas BMI showed limited association within this population. A composite approach integrating relative strength and functional power may provide a more comprehensive characterization of muscle performance relative to muscle quantity than traditional anthropometric measures.

## 1. Introduction

Obesity is a complex and heterogeneous chronic disease characterized not only by excess adiposity but also by profound alterations in body composition, skeletal muscle physiology, and physical function. Although body mass index (BMI) remains the most widely used measure for classifying obesity, it does not distinguish between fat and lean tissue or adequately reflect the heterogeneity in body composition, metabolic health, and functional status among individuals with obesity [1,2]. Recent reviews have reinforced these limitations, emphasizing that BMI fails to capture important differences in ectopic fat accumulation and obesity phenotypes [3]. Consequently, individuals with similar BMI values may differ substantially in body composition, physical function and health risk, highlighting the need for more informative markers of obesity phenotype.

Skeletal muscle dysfunction has emerged as a key contributor to obesity-related morbidity. Beyond increasing mechanical load, excess adiposity may adversely affect muscle structure and function through multiple biological pathways, including chronic low-grade inflammation, insulin resistance, oxidative stress, mitochondrial dysfunction, impaired muscle protein metabolism and ectopic lipid accumulation within skeletal muscle [4,5,6]. These alterations may compromise muscle contractile efficiency and neuromuscular performance, leading to reductions in strength and physical function despite preserved or even elevated absolute muscle mass. Such findings have contributed to growing recognition that muscle quantity alone provides an incomplete characterization of muscle health, particularly in obesity-related conditions such as sarcopenic obesity [7,8].

In this context, the concept of muscle quality (MQ) has gained increasing attention. Although no universally accepted definition exists, MQ is generally defined as the capacity of skeletal muscle to generate force, power, or physical performance relative to its size or quantity [5,9,10]. Recent conceptual reviews further recognize that MQ encompasses a multidimensional construct integrating structural, metabolic, neural and compositional characteristics that determine muscle performance and clinical outcomes [3,10]. Lower levels of muscle quality have been associated with frailty, mobility limitations, disability, metabolic dysfunction, loss of independence and mortality across diverse populations [9,11,12]. Accordingly, contemporary frameworks for sarcopenia and muscle health emphasize muscle strength and physical performance over muscle mass, given their stronger associations with adverse outcomes [12,13]. Recent consensus statements likewise advocate multidimensional assessments of muscle health rather than relying on muscle quantity alone [3,14].

Recent advances in the field have further highlighted that declines in muscle strength and physical performance often occur earlier and progress more rapidly than reductions in muscle mass, a phenomenon known as dynapenia [15]. This dissociation between muscle quantity and function suggests that assessments based exclusively on body composition may fail to identify clinically meaningful impairments in muscle health. Consequently, there is growing interest in developing multidimensional approaches that integrate complementary aspects of muscle function and provide a more comprehensive characterization of the muscle-quality construct [10,15].

Most previous studies have assessed MQ using isolated indicators, such as handgrip strength normalized for body size or muscle mass, imaging-derived estimates of intramuscular adipose tissue, or measures of muscle density and composition. However, skeletal muscle function is inherently multidimensional, encompassing distinct physiological domains, including maximal force production, power generation, neuromuscular coordination and functional performance [9,10]. Recent reviews recommend combining complementary measures of muscle function, as no single parameter adequately captures the complexity of muscle quality [3,16]. In this context, muscle power may decline earlier and more rapidly than muscle strength during ageing, and is strongly associated with mobility and functional independence, suggesting that integrating strength and power provides a more comprehensive assessment of muscle health than either measure alone [11,12]. Furthermore, upper- and lower-limb muscle function reflects partially distinct physiological domains and may decline differently across ageing and obesity, supporting the integration of complementary functional measures when characterizing muscle quality.

Severe obesity is a particularly relevant setting for investigating MQ. Individuals with severe obesity frequently exhibit marked alterations in body composition, increased adipose infiltration of skeletal muscle, reduced physical performance, and elevated risk of disability, yet substantial heterogeneity exists in muscular function and functional capacity [4,6,7]. A more comprehensive characterization of muscle function may contribute to a better understanding of the heterogeneity of musculoskeletal impairment in severe obesity. However, the clinical applicability of composite muscle quality indices remains to be established.

Given the multidimensional nature of MQ, combining complementary functional measures may offer a practical approach for summarizing different aspects of muscle performance relative to muscle quantity [5,9,10]. Previous studies have assessed muscle quality using various functional indices based on relative strength or functional performance [17,18], underscoring the lack of a universally accepted assessment approach. Among these, Barbat-Artigas et al. [17] proposed that muscle quality should be evaluated by considering muscle mass, muscle strength, and muscle power jointly rather than relying on any single component. Consistent with this conceptual framework, combining standardized indicators of upper- and lower-limb muscle function allows complementary domains of muscle performance while preserving clinical interpretability. However, this composite approach has not been formally validated for adults with severe obesity.

In the present study, muscle quality refers to the physiological concept describing muscle function relative to muscle quantity, whereas the composite muscle quality index (Composite MQI) refers specifically to the exploratory composite functional indicator investigated in the present study, calculated from standardized values of handgrip strength normalized by fat-free mass and sit-to-stand power normalized by skeletal muscle mass.

Therefore, the present study aimed to examine the behaviour of an exploratory Composite MQI integrating relative handgrip strength and lower-limb functional power in adults with severe obesity. Additionally, we examined the relationships between the Composite MQI, age, adiposity, and body composition variables in this population. By integrating complementary dimensions of muscle function, we hypothesized that the Composite MQI would provide a practical summary measure of muscle performance relative to muscle quantity for exploratory analyses in adults with severe obesity.

## 2. Materials and Methods

### 2.1. Ethical Concerns

This cross-sectional study was approved by the Ethics Committee of Istituto Auxologico Italiano, IRCCS, Milan, Italy (date of approval: 14 February 2013; reference code: 2013_02_14_09; acronym: QUAMOB) and was performed in compliance with the ethical principles outlined in the Declaration of Helsinki.

### 2.2. Participants

Eligible adults with class II and III obesity (BMI > 35 kg/m^2^), including both men and women aged 18–75 years, who attended the centre between May 2013 and January 2015 were consecutively invited to participate in this cross-sectional study. The study population was recruited at Istituto Auxologico Italiano (IRCCS, Piancavallo-Verbania, Italy), a third-level centre for the rehabilitation and care of severe obesity. Eligible adults attending the centre during the study period were invited to participate. Participants were excluded if they were unable to perform the required functional assessments or had contraindications to bioelectrical impedance analysis, including implanted electronic medical devices (e.g., pacemakers or implantable cardioverter-defibrillators), epilepsy, or pregnancy.

Functional limitations and physical disability that could prevent participation in the study tests were assessed through a brief structured interview adapted from the methodology described by Janssen et al. [19]. Participants were asked about difficulties performing common mobility tasks and activities of daily living, including walking and climbing stairs.

### 2.3. Measurements

#### 2.3.1. Anthropometry and Body Composition

Height was measured to the nearest 0.1 cm using a wall-mounted stadiometer (Wunder Sa.Bi., WU150, Trezzo sull’Adda, Italy), with participants standing barefoot in the orthostatic position, heels together, arms relaxed alongside the body, and head positioned in the Frankfurt horizontal plane. Body weight was measured using a calibrated digital scale (Salus, Milan, Italy) with participants wearing light clothing and no shoes. Body mass index (BMI) was calculated as body weight (kg) divided by height squared (m^2^).

Lower-limb length was measured with the participant standing upright using a flexible, non-elastic measuring tape. The distance between the greater trochanter of the femur and the lateral malleolus was measured on the dominant limb and recorded to the nearest 0.1 cm. This measurement was used to calculate lower-limb muscle power.

Body composition was evaluated using multifrequency tetrapolar bioelectrical impedance analysis (BIA) (Human-IM Scan, DS-Medigroup, Milan, Italy), which applies an alternating current of 800 µA at 50 kHz.

Before the BIA assessment, participants rested in the supine position for 20 min. After the resting period, the skin was cleaned, and four surface electrodes were placed according to the standard tetrapolar whole-body configuration on the right side of the body. The current-injecting electrodes were positioned distally on the dorsal surface of the hand and foot, near the metacarpophalangeal and metatarsophalangeal joints, respectively. The voltage-sensing electrodes were positioned proximally at the wrist, between the radial and ulnar styloid processes, and at the ankle, between the medial and lateral malleoli. During the measurement, participants remained still in the supine position with the arms slightly abducted from the trunk and the legs slightly separated [20].

The BIA measurement was performed using the automated acquisition cycle of the device and lasted approximately 15 s. The raw resistance value at 50 kHz was recorded, and the resistance index at 50 kHz, calculated as height^2^/resistance, was used as an indicator of body composition [20], as it represents the body’s conductive volume, largely determined by total body water and consequently associated with fat-free mass (FFM) [20,21]. Fat-free mass (FFM) was determined using the equation developed by Gray et al. (1989) [22]. Fat mass FM (kg) was derived as the difference between BM (kg) and FFM (kg). Skeletal muscle mass was subsequently estimated using the Janssen equation based on height, resistance, age, and sex [19].

To ensure optimal measurement accuracy and reproducibility, all procedures were conducted under standardized conditions that influence the validity, reliability, and precision of bioelectrical impedance measurements.

#### 2.3.2. Handgrip Strength

Maximal handgrip strength was assessed using a handgrip dynamometer with an adjustable grip (Hand Dynamometer, Lafayette Instrument, Lafayette, IN, USA), following the methodology described by Barbat-Artigas et al. [17]. Participants stood upright and were instructed to exert maximal grip force for at least 4 s. Three trials were performed alternately with each hand, with 10–20 s rest intervals between attempts. Participants received standardized verbal encouragement during each trial to ensure maximal effort. The mean of the six measurements (three trials per hand), expressed in kilograms (kg), was used for statistical analysis.

#### 2.3.3. Sit-to-Stand Test

Lower-limb functional performance was assessed using the 10-repetition sit-to-stand (STS) test, following the protocol described by Takai et al. [23]. Participants were seated on a standardized chair (seat height: 0.42 m; seat depth: 0.45 m) without using the upper limbs for support. At the examiner’s verbal command, they were instructed to stand up to a fully upright position and return to the seated position as rapidly as possible, completing 10 consecutive repetitions. Participants were required to achieve full knee and hip extension during each standing phase and to make complete contact with the seat before initiating the next repetition.

Before formal testing, participants completed familiarization trials at submaximal effort to ensure correct positioning and understanding of the procedure. Two maximal trials were then performed, separated by a 1 min recovery period, and the shortest completion time was retained for analysis. Total execution time was recorded with a digital stopwatch to the nearest 0.1 s, starting on the examiner’s command (“Go”) and ending when the participant reached the fully upright position after the tenth repetition.

The 10-repetition STS test was selected because it provides a practical assessment of lower-limb functional performance and allows estimation of muscle power under dynamic conditions, both of which are important components of muscle quality.

#### 2.3.4. Skeletal Muscle Mass

Skeletal muscle mass (MM) was estimated by whole-body bioelectrical impedance analysis (BIA). Skeletal muscle mass was calculated according to the equation proposed by Janssen et al. [19]:

MM (kg) = [((Height^2^/Resistance) × 0.401) + (sex × 3.825) + (age × −0.071)] + 5.102

where height was expressed in centimetres, resistance was measured by BIA in ohms, sex was coded as 1 for men, and 0 for women, and age was expressed in years.

#### 2.3.5. Lower-Limb Muscle Power

Lower-limb muscle power during repeated sit-to-stand movements (10 consecutive repetitions performed at maximal execution speed) was estimated using the equation proposed by Takai et al. [23]:

$$\mathrm{Muscle}\;\mathrm{Power}\;\left(W\right)\;=\;\frac{\left(L-H\right)\times \mathrm{body}\;\mathrm{weight}\times 9.81\;\times \;10}{\mathrm{Time}}$$

where L represented lower-limb length (distance from the greater trochanter to the lateral malleolus) in metres, H represented chair height in metres, body weight was expressed in kilograms, and time corresponded to the best duration required to complete the 10 repetitions, measured with a stopwatch and expressed in seconds.

Trained investigators performed all anthropometric and functional assessments in accordance with standardized operating procedures to ensure measurement consistency.

### 2.4. Statistical Analysis

Continuous variables were expressed as mean ± standard deviation (SD), whereas categorical variables were presented as absolute and relative frequencies. Data distribution was assessed using the Shapiro–Wilk test, together with visual inspection of histograms and quantile–quantile plots.

Missing data were minimal (<1% for all variables included in the analyses). Therefore, complete-case analyses were performed without data imputation. One male participant was excluded because of incomplete functional assessment data.

Participants were additionally categorized into quartiles according to the Composite MQI for descriptive purposes. This secondary analysis was performed to facilitate the visualization and clinical interpretation of age differences across increasing levels of muscle quality. In contrast, all primary inferential analyses treated the Composite MQI as a continuous variable.

Muscle quality was assessed using both isolated and composite indices reflecting muscle function relative to muscle quantity. Relative handgrip strength normalized by fat-free mass (HG/FFM) and sit-to-stand power normalized by skeletal muscle mass (STS power/MM) were calculated as indicators of upper- and lower-limb muscle quality, respectively, following current conceptual frameworks of muscle quality assessment [5,9,17,18,24]. To obtain a multidimensional measure of muscle quality, a composite muscle quality index (Composite MQI) was calculated as the arithmetic mean of the standardized z-scores of HG/FFM and STS power/MM [20]:

$$\mathrm{Composite}\;\mathrm{MQI}\;=\;\frac{Z\left(\mathrm{HG}/\mathrm{FFM}\right)\;+Z\left(\mathrm{STS}\;\mathrm{power}/\mathrm{MM}\right)}{2}$$

where *Z* denotes the standardized (z-score) value of each component.

Because HG/FFM and STS power/MM are expressed in different units and exhibit different variances, both variables were standardized as z-scores before computing the Composite MQI. Standardization places both measures on the same scale (mean = 0; SD = 1), ensuring that neither component disproportionately influences the composite score because of its measurement scale. Equal weighting was selected a priori because muscle quality is currently considered a multidimensional construct, and there is no established evidence supporting a greater contribution of one functional domain over another [5,9,10]. The Composite MQI was therefore intended to provide a simple, transparent, and clinically interpretable summary measure integrating complementary dimensions of muscle function.

Additional analyses were performed to directly compare HG/FFM, STS power/MM, and the Composite MQI with respect to their associations with age, BMI, and body-fat percentage, and to assess whether the composite provides additional information beyond its strongest component, using multivariable regression models adjusted for age, sex, and BMI. These results are presented in Supplementary Table S1.

Because the objective was not to investigate effect modification, no age × sex interaction term was included in the primary regression model. Instead, sex was included as an a priori adjustment variable. To evaluate the robustness of the proposed Composite MQI, a sensitivity analysis was performed by recalculating the standardized component scores separately within each sex. The resulting sex-standardized Composite MQI was compared with the pooled-standardized Composite MQI using correlation and multivariable regression analyses (Supplementary Table S3).

Another sensitivity analysis evaluated the determinants of estimated skeletal muscle mass. As expected, skeletal muscle mass estimated by the Janssen equation was strongly associated with sex (standardized β = 1.74, *p* < 0.001) and, to a lesser extent, age (standardized β = −0.12, *p* < 0.001), confirming the mathematical properties of the prediction equation rather than an unexpected feature of the composite indicator (Supplementary Table S4).

Conceptually, the Composite MQI is better characterized as a formative composite indicator than as a reflective psychometric scale. Consequently, internal consistency coefficients are descriptive rather than validation criteria [25]. Then, exploratory measures of internal consistency were calculated only to characterize the relationship between its two components and were not used as criteria for index construction or validation. The two components of the Composite MQI showed a weak but statistically significant correlation (Pearson’s r = 0.24, *p* < 0.001), yielding a standardized Cronbach’s α of 0.39 and an equivalent Spearman–Brown coefficient of 0.39.

Between-sex comparisons were performed using Welch’s independent *t*-tests due to unequal variances between groups. Effect sizes were estimated using Cohen’s d. Wilcoxon rank-sum tests were additionally performed as sensitivity analyses and demonstrated similar results.

Associations between continuous variables were assessed using Pearson correlation coefficients. Given the large sample size and approximately symmetric distributions of the primary variables, Pearson correlations were considered appropriate. Correlation strength was interpreted according to conventional Cohen’s thresholds (small: |r| < 0.30; medium/moderate: |r| = 0.30–0.49; large/strong: |r| ≥ 0.50). Correlation analyses were visually represented using a colour-coded heatmap generated with the *corrplot* package in R. Correlations were visually summarized using a colour-coded heatmap generated with the *corrplot* package in R.

Multivariable linear regression analysis was performed to investigate independent predictors of the composite muscle quality index. A parsimonious model including age, sex, and BMI was constructed a priori based on their established relationships with muscle function, body composition, and adiposity. This approach was selected to provide an interpretable model while minimizing multicollinearity and avoiding overadjustment in the absence of additional systematically collected determinants.

As a complementary exploratory analysis, participants were categorized into quartiles based on the Composite MQ index to facilitate descriptive interpretation of the age distribution across groups with relatively lower and higher Composite MQ values. Differences in age across quartiles were assessed using one-way analysis of variance (ANOVA), followed by Tukey’s post hoc tests.

Multicollinearity was assessed using variance inflation factors (VIFs). Statistical significance was established at *p* < 0.05. All analyses were conducted using R statistical software version 4.5.3 (R Foundation for Statistical Computing, Vienna, Austria).

GPT chat (OpenAI), free version, was used for R code refinement.

## 3. Results

### 3.1. Descriptive Analysis

A total of 202 adults with severe obesity were included in the final analysis. The sample presented a mean age of 44.9 ± 14.0 years and a mean BMI of 42.5 ± 5.1 kg/m^2^, characterizing a population with marked excess adiposity. Mean body fat percentage was 46.9 ± 8.0%, whereas mean fat-free mass and muscle mass were 63.6 ± 15.5 kg and 30.5 ± 8.0 kg, respectively. Table 1 summarizes sample characteristics by sex.

The functional assessments (handgrip strength and sit-to-stand muscle power) were used to derive the normalized muscle quality indices (HG/FFM and STS power/MM), which served as the basis for the composite muscle quality index analyzed throughout the study. The proposed composite muscle quality (Composite MQ) index, calculated as the mean standardized score of handgrip strength normalized by fat-free mass (HG/FFM) and sit-to-stand power normalized by muscle mass (STS power/MM), demonstrated approximately normal distribution at the population level.

**Figure 1:**
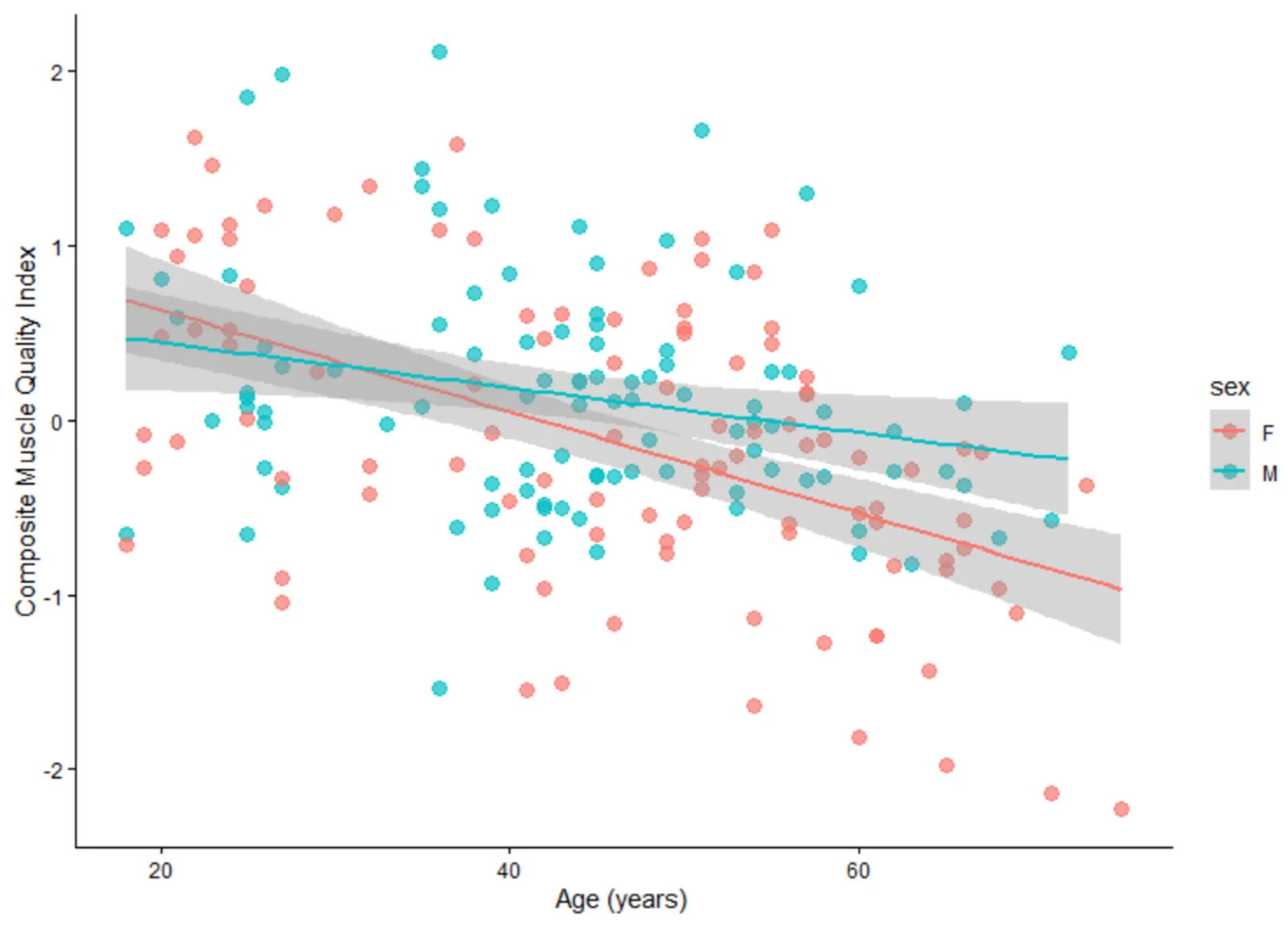
Scatterplot showing the distribution of the composite muscle quality index according to age and sex.

Visual inspection of Figure 1 suggested lower Composite MQI values among older participants, particularly women; however, this descriptive pattern was not formally tested and should be interpreted cautiously. Overall, women had lower Composite MQI values than men (−0.15 ± 0.86 vs. 0.17 ± 0.67; *p* = 0.004), with a small-to-moderate effect size (Cohen’s d = −0.41). The figure provides a descriptive visualization of the relationship between age and Composite MQI values; apparent differences between sexes across age ranges should be interpreted with caution, as age-by-sex interaction effects were not formally tested.

Marked sex differences were observed for absolute handgrip strength and relative handgrip normalized by FFM. Men exhibited substantially higher handgrip values than women (45.0 ± 9.7 kg vs. 23.8 ± 6.9 kg, *p* < 0.001; Cohen’s d = −2.52), as well as higher HG/FFM ratios (0.58 ± 0.12 vs. 0.47 ± 0.12, *p* < 0.001; Cohen’s d = −0.92). In contrast, no statistically significant sex differences were observed in STS power normalized to muscle mass (*p* = 0.4), suggesting relative preservation of lower-limb functional power across sexes after normalization for muscle mass.

### 3.2. Correlation Analyses

**Figure 2:**
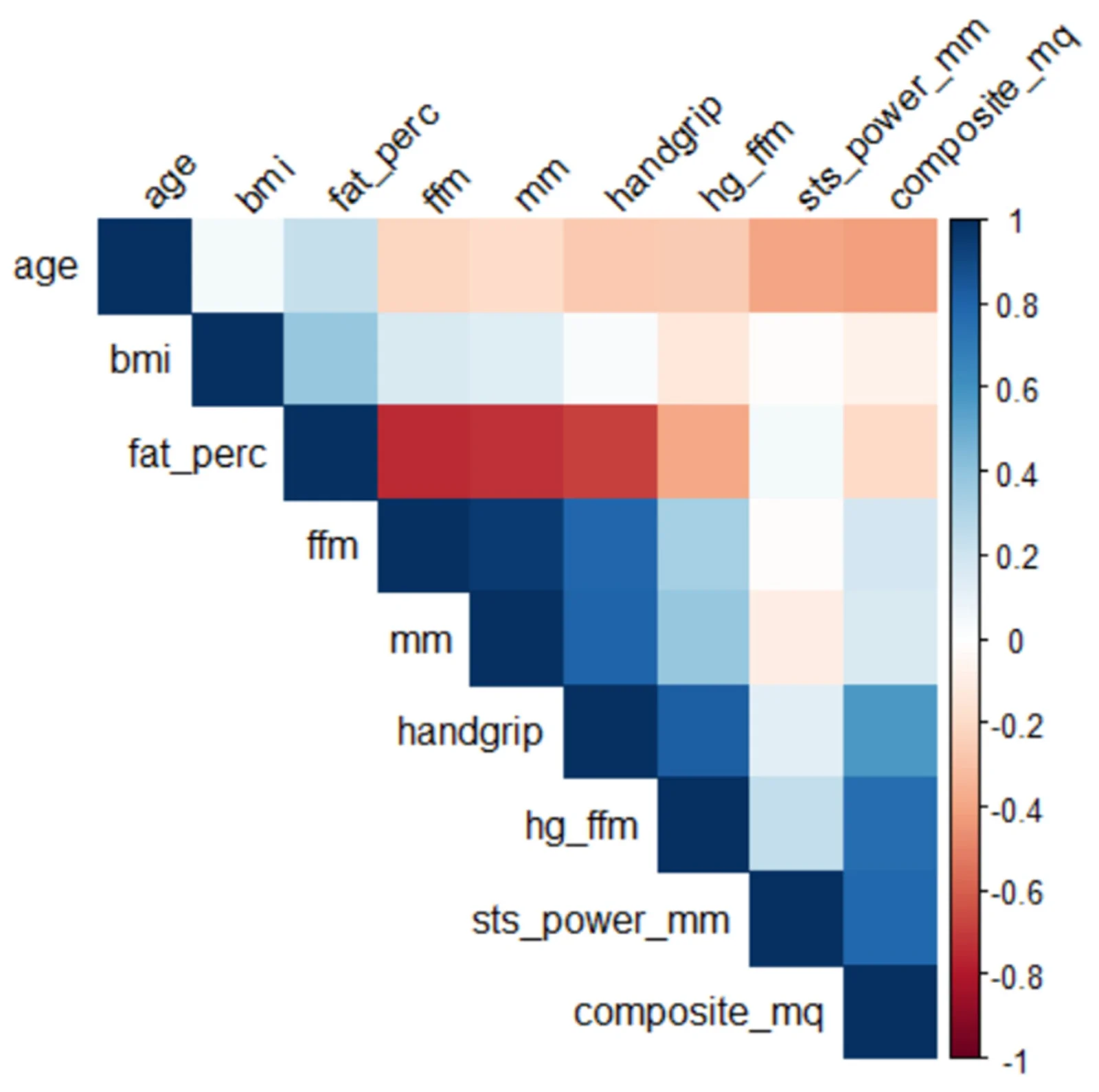
Heatmap of Pearson’s correlation coefficients among age, body mass index (BMI), fat percentage (fat_perc), fat-free mass (FFM), muscle mass (mm), handgrip strength (hg), handgrip strength normalized to hg_FFM (hg/ffm), sit-to-stand power normalized to muscle mass (STS_power_mm), and composite muscle quality index (composite_mq). Positive correlations are shown in blue and negative correlations in red, with colour intensity representing the magnitude of the correlation coefficients.

Correlation analyses revealed significant relationships among adiposity, muscle composition, muscle performance, and the composite muscle quality index (Figure 2).

Age showed a moderate inverse correlation with the composite MQ index (r = −0.41, *p* < 0.001), indicating that older participants tended to have lower Composite MQI values. Age was also negatively correlated with handgrip strength (r = −0.27, *p* < 0.001), HG/FFM (r = −0.25, *p* < 0.001), and STS power/MM (r = −0.40, *p* < 0.001), supporting an inverse cross-sectional association between age and both strength and lower-limb functional performance.

Body fat percentage demonstrated strong inverse correlations with fat-free mass (r = −0.74, *p* < 0.001), skeletal muscle mass (r = −0.73, *p* < 0.001) and absolute handgrip strength (r = −0.69, *p* < 0.001). Higher body fat percentage was also associated with lower HG/FFM (r = −0.40, *p* < 0.001) and lower Composite MQI (r = −0.22, *p* = 0.002). Although body fat percentage was associated with lower Composite MQI, BMI was not. These findings suggest that body composition measures were more closely associated with muscle quality than BMI in this cohort.

Fat-free mass and skeletal muscle mass were strongly correlated with absolute handgrip strength (r = 0.80 and r = 0.810, respectively; both *p* < 0.001). However, their correlations with the Composite MQI were small (FFM: r = 0.205, *p* = 0.003; MM: r = 0.183, *p* = 0.009), suggesting that muscle quantity was only weakly associated with the Composite MQI.

The composite MQ index demonstrated strong positive correlations with HG/FFM (r = 0.79) and STS power/MM (r = 0.79), reflecting the mathematical construction of the multidimensional muscle quality construct. A strong positive association was also observed between composite MQ and absolute handgrip strength (r = 0.60).

BMI was not significantly correlated with the Composite MQI (r = −0.08, *p* = 0.227) or with HG/FFM (r = −0.12, *p* = 0.082) and STS power/MM (r = −0.01, *p* = 0.865), indicating that BMI alone was only weakly related to muscle quality measures in this cohort.

### 3.3. Multivariable Regression Analysis

A parsimonious multivariable linear regression model (Table 2) identified age as the strongest independent cross-sectional predictor of Composite MQI. After adjustment for sex and BMI, higher age remained significantly associated with lower Composite MQI values (β = −0.022, *p* < 0.001). Male sex showed a significant positive association with Composite MQI (β = 0.262, *p* = 0.010), whereas BMI was not independently associated with Composite MQI (*p* = 0.373).

The final model explained approximately 20% of the variability in Composite MQI (adjusted R^2^ = 0.188; model *p* < 0.001; residual standard error = 0.709). Variance inflation factor values were close to 1 for all predictors, indicating the absence of relevant multicollinearity.

An exploratory age-by-sex interaction model yielded borderline statistical significance (*p* ≈ 0.055) and was therefore not retained in the primary model.

### 3.4. Quartile Analysis

To further investigate the clinical relevance of muscle quality, participants were stratified into quartiles according to Composite MQI values. One-way ANOVA demonstrated significant differences in age across MQI quartiles (F = 12.41, *p* < 0.001). Post hoc Tukey analyses (Table 3) revealed that individuals in the highest MQ quartile (Q4) were significantly younger than those in the lowest quartile (Q1), with a mean difference of approximately 15 years (*p* < 0.001). Participants in Q3 were also significantly younger than those in Q1 (*p* = 0.003). These findings indicate that participants with lower Composite MQI values were, on average, older.

**Figure 3:**
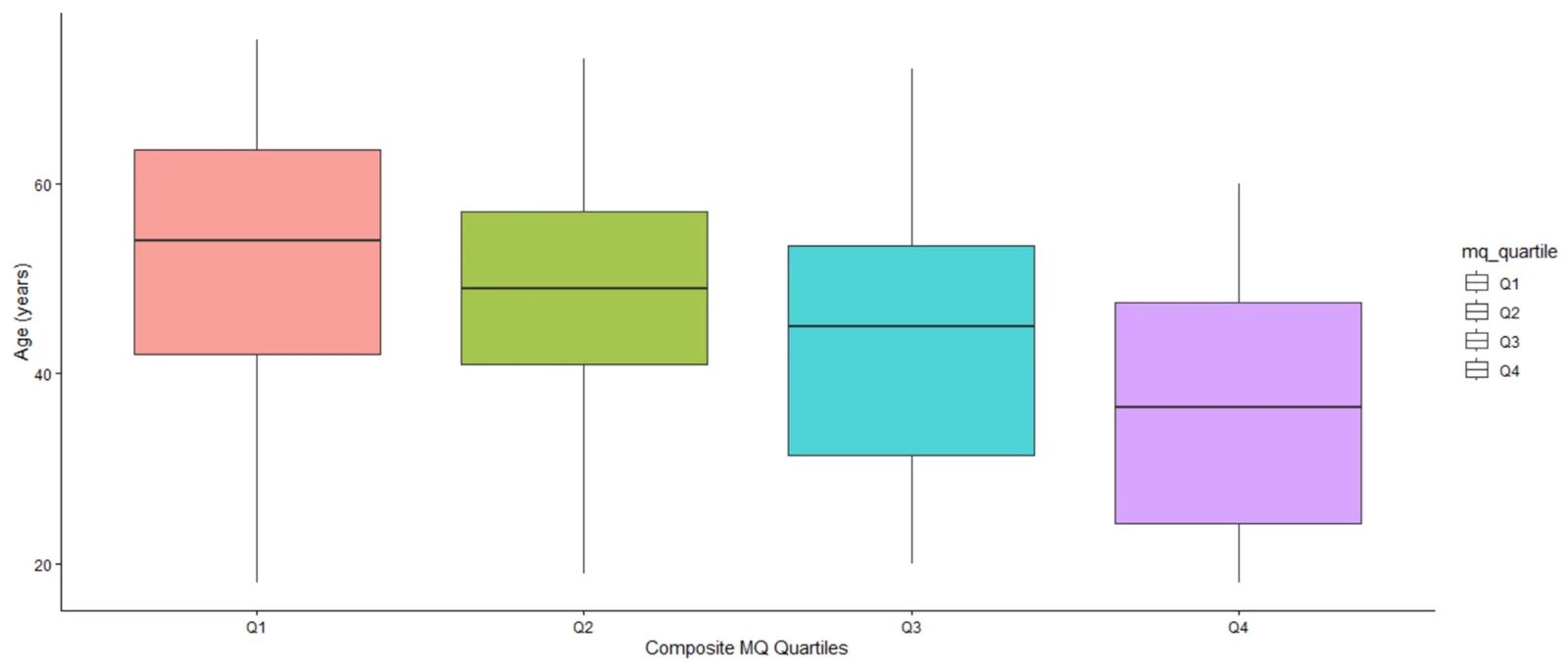
Boxplots illustrating the distribution of age across the four muscle quality (MQ) quartiles. Quartiles are colour-coded as follows: Q1 (pink), Q2 (green), Q3 (blue), and Q4 (purple). The figure shows median values, interquartile ranges, and overall dispersion of age within each MQI category.

The boxplot distribution of age across MQI quartiles (Figure 3) graphically reinforces these findings. Individuals in Q1 (lowest muscle quality) presented the highest median age and the widest age distribution, whereas participants in Q4 (highest muscle quality) were substantially younger. Figure 3 shows how age changes across increasing levels of muscle quality.

Additional comparative analyses demonstrated that the Composite MQI showed a significantly stronger inverse association with age than HG/FFM alone (r = −0.41 vs. −0.25; Williams test, *p* = 0.0002), whereas its correlation with age did not differ from that observed for STS power/MM (r = −0.41 vs. −0.40; *p* = 0.73). Similar patterns were observed in multivariable regression analyses, in which the standardized coefficient for age was larger for the Composite MQI (β = −0.39) than for HG/FFM (β = −0.21) and comparable to STS power/MM (β = −0.41) (Supplementary Table S2).

## 4. Discussion

The present study found that the composite muscle quality index was moderately associated with age, whereas no significant association was observed with BMI, among adults with severe obesity. This finding is consistent with the concept that muscle quality is a multidimensional construct encompassing muscle composition, contractile function, neuromuscular activation, metabolic capacity, and adipose infiltration rather than muscle quantity alone. Recent reviews have emphasized that multidimensional assessments provide a more comprehensive evaluation of muscle health than isolated measures, particularly in obesity and ageing [3,26]. Consistent with this perspective, the proposed Composite MQI, which is an exploratory composite functional indicator that integrates upper-limb strength relative to fat-free mass and lower-limb functional power relative to muscle mass, was designed to provide a multidimensional summary measure of muscle performance relative to muscle quantity, beyond conventional anthropometric indicators.

Overall, muscle quality is considered to reflect not only muscle quantity but also the integrated contributions of structural, metabolic, neural, and functional properties of skeletal muscle [5,9]. By capturing complementary aspects of muscle function, the Composite MQI may provide a broader characterization of muscle performance relative to muscle quantity, consistent with evidence that strength and power are more strongly associated with functional performance and health outcomes than muscle mass alone [6,11,15]. Although age showed the largest observed association with Composite MQI in the present model, the explained variance was modest, suggesting that additional unmeasured biological, clinical, and behavioural factors likely contribute to variability in muscle quality.

The Composite MQI employed in the present study should be interpreted within the context of existing muscle quality assessment approaches. Previous functional indices have primarily relied on isolated measures, such as relative handgrip strength or lower-limb functional performance, to estimate muscle quality relative to muscle quantity [17,18]. While these measures have demonstrated clinical relevance, they each capture only one dimension of skeletal muscle function. Contemporary conceptual frameworks, however, recognize muscle quality as a multidimensional construct encompassing strength, power, neuromuscular performance, and functional capacity [5,9,10]. In this context, the Composite MQI used in the present study represents a practical approach for integrating complementary functional domains into a single interpretable measure, consistent with current recommendations advocating multidimensional assessment of muscle health rather than reliance on isolated functional indicators.

The main finding was the moderate inverse association between age and Composite MQI. Although age-related differences across ages in muscle strength, power, and physical function are well established in the general population [11,12,27], our findings suggest that age-related differences in functional muscle performance are also relevant in severe obesity, even among middle-aged adults. Age showed a larger correlation with the Composite MQI than with muscle quantity alone, indicating that age-related differences in muscle function were more evident than differences in muscle quantity within this cohort. However, the cross-sectional design does not allow conclusions regarding the temporal sequence of these changes.

Although the present study cannot address underlying biological mechanisms, previous studies have reported that advancing age is associated with neuromuscular dysfunction, mitochondrial impairment, anabolic resistance, and intramuscular fat accumulation resulting in declines in strength and physical performance that exceed reductions in muscle mass [3]. Consistent with this view, previous studies have shown that muscle strength and physical performance are only partially explained by muscle quantity, reflecting broader deficits in muscle quality and neuromuscular function [9,11,13]. Recent systematic reviews further demonstrate that muscle strength declines more rapidly than muscle mass and is a stronger predictor of disability, morbidity, and other adverse clinical outcomes [16].

These findings are consistent with the concepts of dynapenia and sarcopenic obesity, which recognize that impairments in muscle strength and physical performance may occur despite preserved muscle mass [8,12,15]. This perspective is reflected in contemporary definitions of sarcopenia and sarcopenic obesity, which increasingly emphasize muscle function over muscle quantity because functional impairments are more strongly associated with disability, morbidity, mortality, and other adverse health outcomes [3,12,13,14].

BMI showed only weak associations with muscle quality indices. Despite the relatively homogeneous and elevated BMI values across the cohort, BMI was neither independently associated with the Composite MQI in multivariable analyses nor meaningfully correlated with functional muscle parameters. These findings suggest that, among adults with severe obesity, BMI alone may have limited ability to capture differences in functional muscle performance.

Unlike body composition measures, BMI does not distinguish between adipose and lean tissue or provide information on muscle function [1,2]. Consequently, individuals with similar BMI values may differ substantially in muscle mass, fat distribution, ectopic fat accumulation, neuromuscular function, and metabolic health, thereby limiting BMI’s ability to capture differences in muscle quality-related functional characteristics [2,7,28]. Recent reviews reinforce the finding that BMI fails to capture the marked heterogeneity of body composition and metabolic phenotypes in obesity, further limiting its usefulness for evaluating muscle quality [3,14].

It should also be acknowledged that the relatively narrow BMI range in this cohort, composed exclusively of adults with severe obesity, may have reduced the ability to detect associations between BMI and the Composite MQI.

The association between adiposity-related measures and lower Composite MQI values is consistent with previous evidence linking excess adiposity to poorer skeletal muscle function beyond its mechanical burden. Previous experimental and clinical studies have identified adiposity-related inflammation, insulin resistance, mitochondrial dysfunction, and myosteatosis as recognized mechanisms that contribute to reduced muscle contractile capacity and neuromuscular efficiency [4,6,29]. Recent evidence further indicates that chronic low-grade inflammation originating from dysfunctional adipose tissue promotes anabolic resistance, oxidative stress, and intramuscular lipid accumulation, accelerating the decline in muscle quality independently of muscle mass [16,26].

These mechanisms have been proposed as contributors to impaired muscle quality in severe obesity, in which muscle mass may be relatively preserved despite substantial impairments in muscle quality and physical function [7,12]. This dissociation between muscle quantity and function is consistent with our findings and may partly explain the observed associations, and reinforces the need for multidimensional assessments of muscle health that extend beyond body size and muscle quantity alone [3,26].

Interestingly, although fat-free mass and muscle mass were strongly associated with absolute handgrip strength, their relationships with the Composite MQ index were considerably weaker. This finding suggests that greater muscle quantity was not necessarily accompanied by better muscle function in this cohort. Similar findings have been reported in ageing and obesity research, supporting the concept of muscle quality as a distinct physiological construct that reflects the functional capacity of skeletal muscle beyond its size or quantity [5,9,10,11,15]. Consequently, assessments based exclusively on muscle mass may underestimate clinically relevant impairments in neuromuscular function and physical performance, supporting current recommendations to prioritize functional measures in the evaluation of muscle health [12].

Sex differences were observed primarily in handgrip strength, with men demonstrating higher absolute and relative values than women. In contrast, lower-limb power normalized to muscle mass did not differ significantly between sexes, suggesting that these differences became less pronounced after accounting for muscle quantity in severe obesity. Previous studies have consistently attributed the greater muscle strength observed in men to differences in muscle mass, muscle fibre characteristics, and endocrine factors [30,31,32]. In the multivariable analysis, male sex was independently associated with higher Composite MQI after adjustment for age and BMI (β = 0.262, 95% CI: 0.06–0.46, *p* = 0.010). This association persisted after accounting for age and BMI. These findings should be interpreted in the context of the marked sex differences observed in absolute handgrip strength and HG/FFM. In contrast, STS power/MM did not differ significantly between sexes. This interpretation is consistent with contemporary evidence indicating that muscle function is influenced by multiple interacting biological and functional factors beyond biological sex alone [3,26,30,31,32].

The marked age gradient observed across MQI quartiles provides additional descriptive information regarding the relationship between age distribution and Composite MQI values. Individuals with the lowest MQI values were approximately 15 years older than those in the highest quartile, suggesting that lower Composite MQI values were observed among older participants and that the index may capture differences in muscle function across the age distribution of this cohort that are not fully reflected by anthropometric or body composition measures. Given the established links among poor muscle quality, mobility limitations, disability, and adverse health outcomes, the Composite MQI may be useful for future studies investigating functional outcomes and intervention responses [6,11,12,13]. The quartile analysis should therefore be interpreted as complementary to the primary continuous-variable analyses rather than as an alternative analytical approach.

Limitations should be acknowledged. First, the cross-sectional design precludes causal inference regarding the observed associations. Second, the study population consisted exclusively of adults with severe obesity recruited from a clinical setting, which may limit the generalizability of the findings. Furthermore, the restricted BMI range resulting from the inclusion of only adults with severe obesity may have limited the ability to detect associations between BMI and the Composite MQI. Although age, sex, and BMI were included in the analyses, other factors that may influence muscle quality, including physical activity, metabolic comorbidities, medication use, nutritional status, and musculoskeletal or neurological conditions, were not systematically assessed, and residual confounding cannot be excluded. Moreover, the observed correlations were predominantly small to moderate in magnitude, indicating that the Composite MQI is influenced by multiple factors beyond those examined in the present analyses. In addition, body composition was estimated using bioelectrical impedance analysis, and potential measurement error in fat-free mass and skeletal muscle mass estimates may have influenced the calculated Composite MQI values. Finally, although the Composite MQI was developed based on current conceptual frameworks of muscle quality, further studies are needed to evaluate its reliability, validity, responsiveness, and applicability across different populations.

Because the Composite MQI is derived from sample-based z-scores, its values are inherently sample-dependent and should not be interpreted against external reference values. Nevertheless, sensitivity analyses using sex-specific standardization yielded virtually identical associations with the main study variables, supporting the robustness of the proposed composite approach while confirming that additional external validation is required before broader clinical application.

An additional limitation is the estimation of skeletal muscle mass. The Janssen bioelectrical impedance equation incorporates both age and sex as predictor variables. Consequently, these variables are mathematically related to one component of the Composite MQI (STS power normalized by skeletal muscle mass), which may have contributed to part of the observed associations. Nevertheless, sensitivity analyses demonstrated that the main findings remained unchanged after alternative standardization procedures, suggesting that the overall conclusions are unlikely to be explained solely by this mathematical dependency.

Despite these limitations, the study also presents important strengths, including a relatively large sample size, the simultaneous assessment of upper- and lower-limb muscle function, and the use of a theoretically informed multidimensional composite index that integrates strength and functional power normalized for muscle quantity.

## 5. Conclusions

In adults with severe obesity, the proposed composite muscle quality index was more strongly associated with age than with BMI, consistent with the view that anthropometric measures alone may not fully characterize muscle function in this condition. By integrating relative upper-limb strength and lower-limb functional power, the Composite MQI provides a theoretically informed multidimensional summary measure of muscle performance relative to muscle quantity. Future longitudinal studies are warranted to determine whether multidimensional muscle quality indices are associated with clinically relevant outcomes and whether they provide additional information beyond traditional measures.

## Figures and Tables

**Table 1:** Demographic, anthropometric, body composition, and muscle function characteristics of women (*n* = 103) and men (*n* = 99) included in the study.

Characteristic	F *n* = 103 ^1^	M *n* = 99 ^1^	95% CI	*p*-Value ^2^
Age (years)	46 ± 15.1	43.7 ± 12.7	42.91–46.80	0.13
BMI (kg/m^2^)	42.8 ± 5.7	42.2 ± 4.4	41.81–43.22	0.9
FFM (kg)	50.2 ± 5.1	77.5 ± 9.2	61.41–65.72	<0.001
FFM (%)	46.3 ± 4.7	60.1 ± 2.7	51.96–54.15	<0.001
Fat (kg)	59.1 ± 13.5	51.8 ± 8.9	53.85–57.19	<0.001
Fat (%)	53.5 ± 5.3	40.1 ± 3.2	45.84–48.06	<0.001
Handgrip (kg)	23.8 ± 6.9	45 ± 9.7	32.32–36.08	<0.001
HG_FFM	0.47 ± 0.1	0.58 ± 0.1	0.51–0.55	<0.001
STS_best time (s)	19.4 ± 8.2	16.8 ± 5.4	17.18–19.15	0.009
STS_Power	263 ± 112	390 ± 143	305.79–345.49	<0.001
STS_Power_MM	11.2 ± 4.8	10.4 ± 3.5	10.22–11.38	0.4
Composite_MQ	−0.15 ± 0.9	0.17 ± 0.7	−0.11–0.11	0.008

^1^ Mean ± SD ^2^ Welch Two-Sample *t*-test. Legend: BMI, body mass index; FFM, fat-free mass; HG, handgrip strength; HG/FFM, handgrip strength normalized by fat-free mass; STS power, sit-to-stand muscle power; STS power/MM, sit-to-stand muscle power normalized by skeletal muscle mass; Composite MQ, composite muscle quality index.

**Table 2:** Multivariable linear regression model used to predict the composite muscle quality index based on selected predictors.

Predictor	β Coefficient	Standard Error	95% CI	t Value	*p* Value
Intercept	1.240	0.451	0.34–2.12	2.75	0.007
Age (years)	−0.022	0.004	−0.03–−0.01	−6.15	<0.001
Male sex	0.262	0.100	0.06–0.46	2.62	0.010
BMI (kg/m^2^)	−0.009	0.010	−0.03–0.01	−0.89	0.373

**Table 3:** Post hoc comparisons according to composite muscle quality (MQ) quartiles.

Comparison *	Mean Difference (Years)	95% CI	Adjusted *p*
Q2 vs. Q1	−4.22	−10.88 to 2.45	0.359
Q3 vs. Q1	−8.98	−15.64 to −2.32	0.003
Q4 vs. Q1	−14.98	−21.68 to −8.28	<0.001
Q3 vs. Q2	−4.76	−11.43 to 1.90	0.252
Q4 vs. Q2	−10.77	−17.46 to −4.07	<0.001
Q4 vs. Q3	−6.00	−12.70 to 0.70	0.097

* Tukey post hoc comparisons.

## Data Availability

Raw data will be uploaded to www.zenodo.org. The datasets generated and analyzed during the current study are available from the corresponding author upon reasonable request. Because the dataset contains individual clinical information collected at a tertiary obesity centre, public deposition is restricted in accordance with institutional ethical approval and data protection regulations.

## Supplementary Materials

The following supporting information can be downloaded at: https://www.mdpi.com/article/10.3390/jcm15176696/s1, Table S1. Correlations and Multivariate Regression Analysis. Table S2. Sensitivity analysis. Table S3: Sensitivity analysis evaluating the influence of sex-specific standardization on the Composite Muscle Quality Index. Table S4: Determinants of estimated skeletal muscle mass (Janssen equation).

## Abbreviations

The following abbreviations are used in this manuscript:

BIA	Bioelectrical impedance analysis
BMI	Body mass index
FFM	Fat-free mass
HG	Handgrip strength
MM	Skeletal muscle mass
MQ	Muscle quality
STS	Sit-to-stand
Composite MQI	Composite Muscle Quality Index
