# Supplementary material for: A Multidimensional Muscle Quality Index in Severe Obesity: Relationships with Ageing, Adiposity, and Functional Performance
Source: J Clin Med. 2026 Aug 29;15(17):6696. doi: 10.3390/jcm15176696 (PMC13566254; doi:10.3390/jcm15176696)
Supplement: Supplementary file 1 [file jcm-15-06696-s001.zip › jcm-4485743-supplementary.pdf]

Supplementary Table S1. Correlations and Multivariate Regression Analysis

Outcome	Age r	BMI r	Fat % r	β Age	Adjusted R^2
HG/FFM	-0.25	-0.12	-0.39	-0.21	0.220
STS power/MM	-0.40	-0.01	0.05	-0.41	0.161
Composite MQI	-0.41	-0.09	-0.22	-0.39	0.188

Legend: Differences between dependent correlations were tested using the Williams test implemented in the R cocor package.

Supplementary Table S2. Sensitivity analysis.

Outcome	Pooled standardization	Sex-specific standardization
Correlation with age	-0.410	-0.392
Correlation with BMI	-0.085	-0.070
β age (adjusted)	-0.39	-0.39
β BMI (adjusted)	-0.06	-0.05
β sex (adjusted)	0.17	-0.03
R^2	0.200	0.157
Correlation between MQI versions	—	**0.976**

Supplementary Table S3: Sensitivity analysis evaluating the influence of sex-specific standardization on the Composite Muscle Quality Index

Outcome	Original Composite MQI	Sex-standardized Composite MQI
β Age	-0.39	-0.39
p Age	<0.001	<0.001
β Sex	0.33	-0.03
p Sex	0.010	0.657
β BMI	-0.06	-0.05
p BMI	0.373	0.424
R^2	0.200	0.157

Legend: Comparison between pooled and sex-specific standardization demonstrates that the inverse association between age and the Composite MQI remained essentially unchanged, whereas the sex effect disappeared after within-sex standardization, indicating that pooled standardization primarily influenced between-sex differences but did not materially affect the age-related association.

Supplementary Table S4: Determinants of estimated skeletal muscle mass (Janssen equation)

Variable	β	95% CI	p
Age	-0.12	-0.102 - -0.032	<0.001
Sex	1.74	12.959 – 14.928	<0.001
BMI	0.18	0.191 – 0.385	<0.001

Legend: As expected, skeletal muscle mass estimated by the Janssen equation was strongly associated with sex and, to a lesser extent, age, reflecting the variables incorporated into the prediction equation.
